# Novel Bacterial Topoisomerase Inhibitors Exploit Asp83 and the Intrinsic Flexibility of the DNA Gyrase Binding Site

**DOI:** 10.3390/ijms19020453

**Published:** 2018-02-03

**Authors:** Sebastian Franco-Ulloa, Giuseppina La Sala, Gian Pietro Miscione, Marco De Vivo

**Affiliations:** 1 COBO Computational Bio-Organic Chemistry Bogotá, Chemistry Department, Universidad de los Andes, Cra 1 No 18A-12, 111711 Bogotá, Colombia; sebastian.franco@iit.it; 2 Laboratory of Molecular Modeling and Drug Discovery, Istituto Italiano di Tecnologia, via Morego 30, 16163 Genova, Italy; giuseppina.lasala@bikitech.com; 3 IAS-5/INM-9 Computational Biomedicine Forschungszentrum Jülich Wilhelm-Johnen-Straße, 52428 Jülich, Germany

**Keywords:** DNA gyrase, NBTI, topoisomerases, drug resistance, MD simulation, docking, antibiotics

## Abstract

DNA gyrases are enzymes that control the topology of DNA in bacteria cells. This is a vital function for bacteria. For this reason, DNA gyrases are targeted by widely used antibiotics such as quinolones. Recently, structural and biochemical investigations identified a new class of DNA gyrase inhibitors called NBTIs (i.e., novel bacterial topoisomerase inhibitors). NBTIs are particularly promising because they are active against multi-drug resistant bacteria, an alarming clinical issue. Structural data recently demonstrated that these NBTIs bind tightly to a newly identified pocket at the dimer interface of the DNA–protein complex. In the present study, we used molecular dynamics (MD) simulations and docking calculations to shed new light on the binding of NBTIs to this site. Interestingly, our MD simulations demonstrate the intrinsic flexibility of this binding site, which allows the pocket to adapt its conformation and form optimal interactions with the ligand. In particular, we examined two ligands, AM8085 and AM8191, which induced a repositioning of a key aspartate (Asp83B), whose side chain can rotate within the binding site. The conformational rearrangement of Asp83B allows the formation of a newly identified H-bond interaction with an NH on the bound NBTI, which seems important for the binding of NBTIs having such functionality. We validated these findings through docking calculations using an extended set of cognate oxabicyclooctane-linked NBTIs derivatives (~150, in total), screened against multiple target conformations. The newly identified H-bond interaction significantly improves the docking enrichment. These insights could be helpful for future virtual screening campaigns against DNA gyrase.

## 1. Introduction

DNA gyrase is a metalloenzyme that belongs to the topoisomerase family. This family of proteins manipulates the topological state of DNA [1,2]. The DNA gyrase is chiefly present in prokaryotes [3], where it catalyzes the cleavage of the two helices of a DNA strand in an ATP-dependent manner, making it essential for bacterial survival [4]. DNA gyrase is a validated pharmaceutical target for antibiotics [5], and some of its homologous human forms are targeted by several anticarcinogens [6,7].

**Figure 1:**
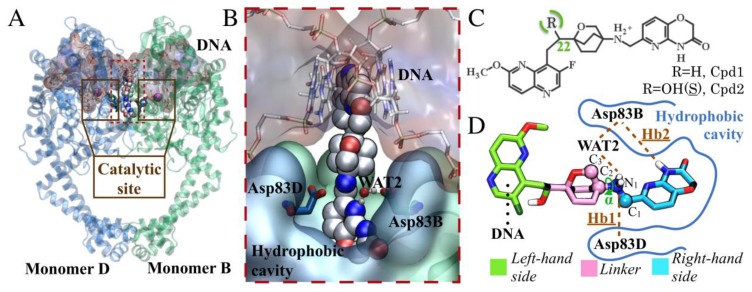
(**A**) Representation of the GyrA-GyrB truncated core fusion (PDB 4PLB). This structure contains monomer B and monomer D, which are shown in green and blue, respectively. The DNA is represented as red surface. The two maroon boxes enclose the protein’s catalytic site. AM8191 (Cpd2) is displayed in white carbons, WAT2 in CPK representation, and Mg^2+^ as purple spheres; (**B**) Binding mode of Cpd2 into DNA gyrase; (**C**) 2D representation of the NBTIs studied, Cpd1 and Cpd2, in their protonated state, as in our simulations; (**D**) Scaffold classification for Cpd2, showing the left-hand side (LHS) in green, the linker in pink, and the right-hand side (RHS) in light blue.

DNA gyrase comprises two subunits, GyrA and GyrB, which form the A2B2 tetramer in the active enzyme [8]. GyrA contains the catalytic tyrosine, which can cleave the DNA when correctly positioned adjacent to the GyrB TOPRIM domain (Figure 1) [9]. Quinolones are drugs for treating bacterial infections [10,11]. They bind to the cleavage binding site and poison the DNA gyrase by stabilizing the cleaved DNA–protein complex [12]. Quinolone derivatives have been used for decades as antibiotics of last resort [13]. However, their excessive use and misuse have led to alarming rates of quinolone-resistant bacteria strains [14]. Coumarin derivatives, such as novobiocin, are another class of DNA gyrase inhibitors [15]. Unlike quinolones, coumarins target the ATP binding pocket located in the GyrB subunits, and they are active against the quinolone-resistant form of the enzyme [16]. However, the coumarin derivatives manufacture has been halted due to the presence of safer and more effective agents [17].

Recently a number of papers have been published [18,19,20,21] on the so-called “novel bacterial topoisomerase inhibitors” (NBTIs) [22]. Structural studies demonstrated that these compounds inhibit DNA gyrase by binding to a newly identified pocket located at the interface between the two GyrA subunits, stabilizing the pre-cleaved DNA–protein complex [23]. Since NBTIs target a new site of the DNA gyrase structure, they may not suffer from cross-resistance with quinolones or coumarin-derivatives [24]. This makes the development of NBTIs a promising strategy for identifying potent antibacterial agents active against multidrug-resistant strains of bacteria, such as methicillin-resistant *Staphylococcus aureus* (MRSA) [25].

In more detail, the chemical scaffold of NBTIs comprises two heterocycles connected via an aliphatic linker, usually containing a basic nitrogen (Figure 1D) [26]. Structural data have shown that NBTIs interact via hydrophobic contacts with the target, forming π–π interactions between two bases of the DNA. The linker portion also establishes a conserved H-bond with the side chain of Asp83 of monomer D (Asp83D) at the binding site (PDB 4PLB, Figure 1B) [21,23,27,28,29,30].

In this work, we performed classical molecular dynamics (MD) simulations of a truncated core fusion of GyrA and GyrB in complex with either AM8085 or AM8191, which are two potent NBTIs [21]. Our goal was to identify and characterize the key drug–target interactions required for drug binding by means of MD simulations. We observed that the intrinsic flexibility of the NBTI binding pocket allows the formation of an additional crucial H-bond between the NBTI and Asp83 from monomer B (Asp83B, with the same nomenclature as in the 4PLB crystal structure). These findings were subsequently validated by docking calculations of ~150 NBTI cognates bearing a common oxabicyclooctane linker [21,30,31,32,33,34], which were docked into the target in different conformations. Our results confirm that the newly identified H-bond with Asp83B is important in favoring the tight binding of NBTIs to the recently identified pocket of DNA gyrase.

## 2. Results and Discussion

### 2.1. MD Simulations for NBTI Binding

In the present work, we began by performing a comparative analysis of three model systems investigated via molecular dynamics (MD) simulations of ~100 ns each. System 1 (Sys1) comprises the truncated core fusion of DNA gyrase in complex with the inhibitor AM8085 (Cpd1 in Figure 1C; IC_50_ = 0.22 μM against *Staphylococcus aureus* DNA gyrase). System 2 (Sys2) is the same enzyme in complex with the inhibitor AM8191 (Cpd2 in Figure 1C; IC_50_ = 1.02 μM against *Staphylococcus aureus* DNA gyrase). Notably, the two inhibitors share the same oxabicyclooctane chemical scaffold, differing only in the presence of an OH group in Cpd2, at position 22 (Figure 1C). For comparison, we also considered the apo form of the core (SysAPO). See Section 3 for details.

**Figure 2:**
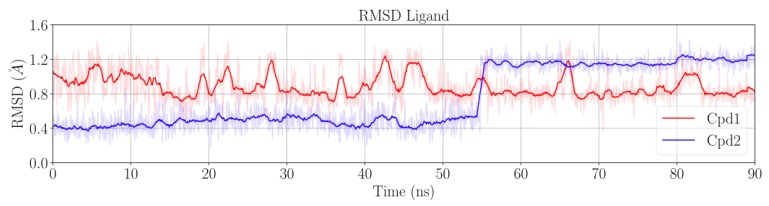
Time evolution of the root-mean square deviation (RMSD) of Cpd1 (**red**) and Cpd2 (**blue**) computed on Sys1 and Sys2 trajectories, respectively.

After the equilibration phase (~10 ns), the protein and the DNA were stable. The RMSD values were ~1.6 ± 0.1 Å for the protein in both Sys1 and Sys2, and ~1.0 ± 0.1 Å for DNA in both Sys1 and Sys2 (Figure S1). Cpd1 was also stable for the entire simulation, with an RMSD of ~0.9 ± 0.2 Å (Figure 2). Conversely, Cpd2 remained very stable for only the first ~55 ns of the trajectory, showing a low RMSD value of ~0.5 ± 0.1 Å. Then, the RMSD suddenly increased to ~1.2 ± 0.1 Å, suggesting a conformational rearrangement that was firmly maintained until the end of the simulation (Figure 2).

The crystallographic H-bond formed by Asp83D and the basic nitrogen of the linker (hereafter referred to as Hb1, Figure 1D) was already present in the starting X-ray structure. During our simulations, we observed that this H-bond was stably maintained during the entire simulation (persistency of 98% in both Sys1 and Sys2). Notably, Hb1 is present in all the available DNA gyrase/NBTI complexes, suggesting its relevance for target–ligand interaction and binding [21,23,27,28,29,30].

**Figure 3:**
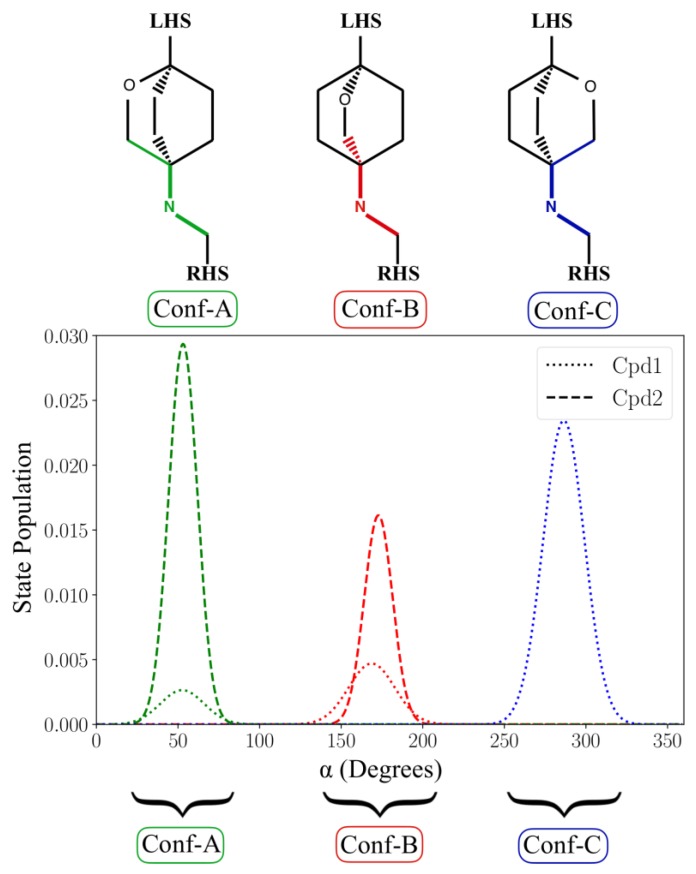
State population of the value of the α dihedral angle for both Cpd1 and Cpd2 computed over the simulated time. For Cpd1, the α dihedral angle populates the three conformational states, Conf-A, Conf-B, and Conf-C. Cpd2 visits only Conf-A and Conf-B.

Interestingly, in both Cpd1 and Cpd2, we observed the free rotation of the oxabicyclooctane ring during the MD simulations. In theory, the oxabicyclooctane ring can adopt three different conformations, Conf-A, Conf-B, and Conf-C. These conformations are identified by three different values of the dihedral angle α formed by the atoms C_1_-N_1_-C_2_-C_3_ (Figure 1D and Section 3). In the case of Cpd1, α rotates continuously, visiting the crystallographic Conf-A for 8.9% of the simulated time, and Conf-B and Conf-C for the remaining 16.6% and 74.5%, respectively (Figure 3). Conversely, for Cpd2, the dihedral angle α is more stable, and the inhibitor remains in the crystallographic conformation Conf-A for the initial ~55 ns, as also indicated by its low RMSD (Figure 2 and Figure 3). Then, α rotates, and the molecule assumes Conf-B, where it remains until the end of the simulation. Notably, Cpd2 never adopted Conf-C (Figure 3). This is due to the presence of the hydroxyl group in the linker, which allows the formation of a water-mediated intramolecular H-bond with the oxygen of the oxabicyclooctane (WAT1 in Figure S2). This water-mediated intramolecular H-bond stabilizes Conf-B, decreasing the conformational fluctuations of the linker, and preventing the formation of Conf-C. 

**Figure 4:**
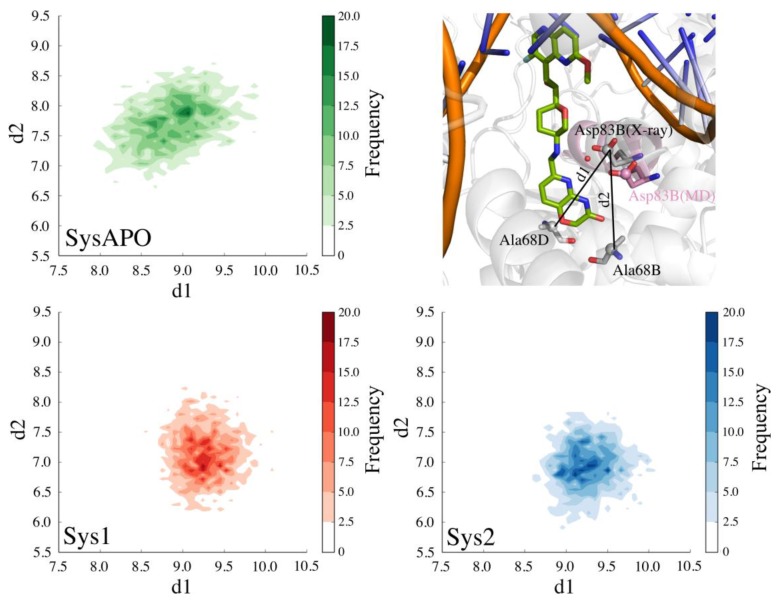
2D plot of SysAPO (**green**), Sys1 (**red**), and Sys2 (**blue**) MD trajectory data plotted along d1 (distance between the Cγ of Asp83B and the Cα of Ala68D) and d2 (distance between the Cγ of Asp83B and the Cα of Ala68B).

A second water molecule (WAT2, Figure 1D) is also present in several of the available X-ray structures (PDB codes: 4PLB, 2XCS, 4BUL, 5IWI, 5NPP) [21,23,27,28,29]. This water molecule resides at the entrance of the hydrophobic cavity in the enzyme and participates in a water-mediated H-bond interaction between Asp83 of chain B (hereafter referred to as Asp83B) and the NBTIs’ linker (Figure 1D). In both Sys1 and Sys2, our MD simulations show that WAT2 leaves the binding site during the unrestrained equilibration, suggesting that this water molecule is poorly stable in the protein–ligand complex. However, the displacement of WAT2 preludes the formation of a new H-bond (hereafter referred to as Hb2) between Asp83B and the nitrogen of the pyrido-oxazinone ring of the RHS portion of the inhibitor (Figure 1D). In fact, Hb2 has an overall occupancy of 99% in both Sys1 and Sys2. Notably, Asp83B’s side chain changes its crystallographic position to approach the RHS to form this new H-bond. Importantly, the flexibility of the binding site is a key feature for binding, which has been similarly observed for other proteins [35,36,37]. Here, the conformational displacement of Asp83B was captured by monitoring the distance from the Cγ of Asp83B to the Cα of the nearby residues Ala68D (d1) or to the Cα of Ala68B (d2) (Figure 4). In detail, in both MD simulations of Sys1 and Sys2, d2 shortened from 9.1 Å (i.e., X-ray value) to ~7 Å, while d1 remained close to the crystallographic value (i.e., 9.1 Å). As expected, in the absence of the ligand, Asp83B showed a pronounced flexibility with respect to the holo systems (Sys1 and Sys2). In fact, in the MD simulations of SysAPO, both d1 and d2 varied remarkably with respect to their crystallographic value, indicating that Asp83B sampled a broader conformational space in SysAPO than in Sys1 and Sys2 (Figure 4). These results demonstrate that Asp83B can indeed adopt multiple orientations, in addition to that resolved in 4PLB. Interestingly, the electron density map of 4PLB shows poor electron density for Asp83B, which suggests significant flexibility for this residue, which can therefore flip to adopt different conformations. In addition, to better characterize the possible spatial configurations of Asp83B, we visually inspected 43 X-ray structures of DNA gyrase and topoisomerase IV (Table S1). Through a structural superposition of the X-ray structures, we observed that, in 35 of them, the corresponding Asp83B’s side chain adopts the same orientation as in the X-ray structure used in our study (i.e., 4PLB), where Hb2 is prevented (see Table S1 for details). However, we also found that the corresponding Asp83B is shifted in four structures, assuming an optimal orientation for Hb2 formation, in agreement with our observation from the MD simulations in which the ligand is bound to the target. Intriguingly, in three PDB structures (4Z2C, 4Z2D, 4Z2E) [38], Asp83B points down. In 3IFZ, a major conformational rearrangement is visible, which involves the entire helix, along which Asp83B is located [39]. Taken together, these structural data confirm that Asp83B is indeed very flexible, being able to adopt a variety of conformations, including the one observed in our MD simulations, which is stabilized by the formation of Hb2 with the ligand.

Overall, our MD simulations suggest that both Asp83B and Asp83D are key for the binding of this selection of NBTIs. In detail, we found that Asp83B can modify its conformation to stably form a previously uncharacterized H-bond Hb2 interaction with the right-hand side (RHS) of Cpd1 and Cpd2, while Asp83D locks the NBTIs linker through the crucial H-bond Hb1. That is, these two aspartates act as two arms that clamp the ligands in the pocket, stabilizing their binding. Notably, biochemical studies support the importance of the aspartates in the protein–ligand binding. These studies have demonstrated that NBTIs (e.g., AZ6142, AZ0217, MCHEM18) lose potency when these aspartates are replaced by a glycine [40].

### 2.2. Accounting for Asp83B Flexibility and H-Bond Formation in Docking Calculations

**Figure 5:**
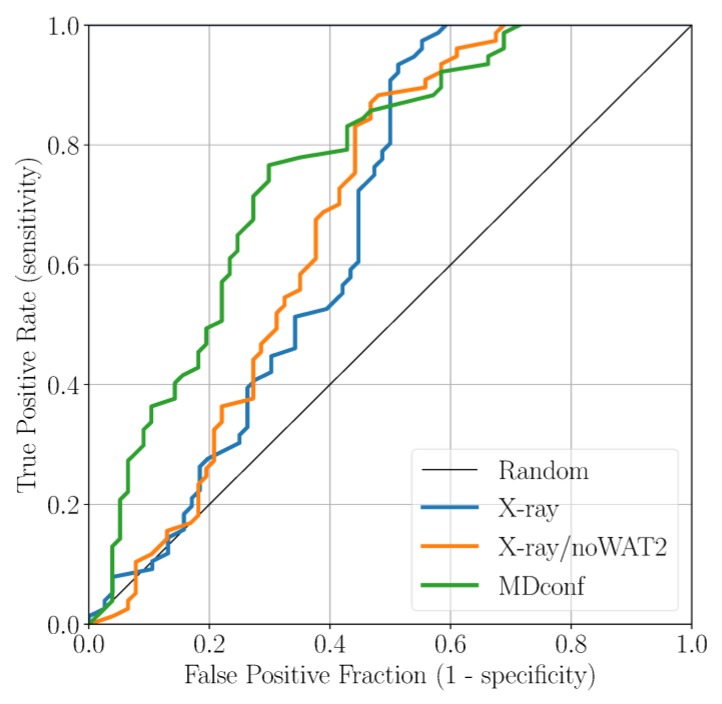
ROC curve for the docking results based on the three receptor structures studied.

Here, we applied docking calculations to validate the outcomes obtained from our classical MD simulations [41,42]. The final goal was to define the optimal docking set-up for virtual screening campaigns of NBTIs against the DNA gyrase. To this end, we first collected 146 cognate compounds belonging to the NBTI class of inhibitors, retrieved from six publications from the same research group [21,30,31,32,33,34]. Notably, these molecules are close analogs of Cpd1 and Cpd2, sharing the same oxabicyclooctane scaffold. These ligands were divided into 83 active and 63 inactive ones, according to their MIC value. In the context of this study, our subjective criterion was to consider as active only those compounds that display an MIC ≤ 1 µg/mL (see Section 3 for details). Subsequently, we docked all the active and inactive ligands against three representative structures of DNA gyrase: (i) the original X-ray structure of the truncated core fusion (referred to as X-ray), (ii) the same structure without WAT2, as found in our MD simulations (referred to as X-ray/noWAT2), and (iii) a representative frame from the MD simulations of Sys2, bearing Asp83B in a conformation prone to forming the newly identified H-bond Hb2 with the ligand (referred to as MDconf). After docking the entire dataset against the three models, we quantified the enrichment by computing both the BEDROC and the ROC curve. This analysis was conducted to understand which docking protocol would better discriminate active from inactive ligands. As a result, using the X-ray structure as receptor, the BEDROC (α = 8.0) value was 0.55 (Figure 5). By excluding the crystallographic water WAT2 (X-ray/noWAT2 system), the resulting BEDROC value of 0.52 is comparable to the previous one (Figure 5). The presence of the WAT2 water molecule, therefore, does not substantially affect the docking results, meaning that it is likely not essential for ligand binding. This is in line with our MD simulations, which show that WAT2 is particularly unstable within the binding site. Remarkably, the enrichment improved when we used MDconf as the receptor. In this case, the BEDROC value increased to 0.72 (Figure 5). Importantly, after visually inspecting the resulting binding poses, we noted that ~70% of the docked active ligands formed Hb2 with Asp83B. This demonstrates that this newly identified interaction is highly favorable for ligand binding at this recently identified site of DNA gyrase. 

## 3. Materials and Methods

### 3.1. Systems Preparation and MD Simulations 

In this study, we performed classical MD simulations of three biomolecular systems: (1) SysAPO, which comprises a GyrA-GyrB truncated core fusion in complex with DNA; (2) Sys1, which comprises ligand AM8085 (Cpd1) in complex with SysAPO, and (3) Sys2, which comprises ligand AM8191 (Cpd2) bound also to SysAPO. SysAPO was modeled starting from PDB code 4PLB [21], with a resolution of 2.69 Å. We removed the co-crystallized ligand (Cpd2) and all the water molecules except for WAT2. Furthermore, the 12-residue loop connecting GyrA and GyrB was modeled using Prime [43,44]. Sys1 was prepared starting from the previously described model, in which Cpd1 was docked using the Glide software [45,46]. Sys2 was prepared in the same way as SysAPO, but this time keeping the native ligand (Cpd2). For the sake of clarity, we specify that in this study we name chains as in the original crystal structure (namely, monomers B and D in PDB code 4PLB), where each monomer is a truncated fusion of GyrA and GyrB subunits. Thus, the letters B and D that indicate the aspartate residues refer to the chain they belong to in the PDB. However, Asp83B (the aspartate belonging to Chain B, as named in the PDB) corresponds to Asp83A according to the standard naming of residues in GyrA. At the same time, Asp83D (the aspartate belonging to Chain D, as named in the PDB) corresponds to Asp83C according to the standard naming of residues in GyrA.

The protein and the DNA were parameterized using AMBER’s ff14SB force field [47]. The partial charges of the ligands (Cpd1 and Cpd2) were calculated with the RESP [48] model using a 6-31G* basis set, and parameterized using the general AMBER force field (GAFF) [49]. Both, Cpd1 and Cpd2 were simulated with their linker’s nitrogen atom protonated. The three systems were immersed in a TIP3P water box [50], leaving 15 Å from the solute and the edges of the box. The resulting box was 136 × 128 × 120 Å^3^.

First, the systems were minimized using the steepest descent method for a maximum of 5000 steps. Then, the systems were gradually heated to reach 300 K in 100 ps, using modified Berendsen thermostats [51] and applying harmonic restraints with force constants of 1000 kJ mol^−1^ nm^−2^ in all three directions to the systems’ backbone and the ligands. The heated systems were subsequently submitted to an equilibration phase of ~200 ps in an NPT ensemble, maintaining the temperature at 300 K and applying a Parrinello–Rahman barostat [52]. At this point, all restraints were removed and the systems were equilibrated for ~10 ns, each. Then, a production phase of ~90 ns was collected, for each of the systems. During the MD simulations, the LINCS algorithm [53] was used to constrain all bonds, and the Particle Mesh Ewald (PME) [54] was used to account for electrostatic interactions. The integration time step was 2 fs. All the MD simulations were carried out with Gromacs version 4.6 (Gromacs, Groningen, Netherlands) [55]. This workflow has already been successfully implemented for similar studies in other biological systems [56,57].

### 3.2. Analysis of the MD Simulations 

We evaluated the stability of the three systems (SysAPO, Sys1, and Sys2) during the MD simulations by computing the root-mean-square deviation (RMSD) of the protein’s backbone using the g_rms tool from Gromacs [55]. The occupancy of H-bonds along the trajectories was computed using the g_hbond tool from Gromacs and the readHBmap script. We considered as an H-bond only those configurations with a donor-acceptor distance less than 3.5 Å and a hydrogen-donor-acceptor angle greater than 30°. The distances and dihedral angles were computed using the g_dist and g_angle tools from Gromacs. To define the three different conformations of the ligands observed during the MD trajectories of Sys1 and Sys2, we used the dihedral angle α formed by the atoms C_1_-N_1_-C_2_-C_3_ (Figure 1). In particular, we divided the α values into three equal intervals (0° to 120°, 120° to 240°, and 240° to 360°), corresponding to Conf-A, Conf-B, and Conf-C, respectively. 

### 3.3. Protein Preparation and Building of the Training Set

For our docking calculations, we used three representative conformations of the GyrA-GyrB truncated core fusion: (i) the 4PLB X-ray structure model including the 12-residue missing loop (X-ray) [21]; (ii) the same model as in (i), stripping the WAT2 water molecule from the binding site (X-ray/noWAT2); (iii) the first frame of the MD simulations of Sys2 where Asp83B’s conformational change occurs and is prone to form Hb2. All three conformations were prepared with the Protein Preparation Wizard’s [58,59] protocol implemented in Maestro. Finally, a restrained minimization was performed, setting a maximum RMSD of 0.3 Å, and a 10 Å edge-long cubic grid was centered in the NBTI binding site.

For this study, we used 126 ligands retrieved from six recent publications from the same group. All 126 compounds share a common oxabicyclooctane scaffold as Cpd1 and Cpd2 [21,30,31,32,33,34]. Notably, we selected only those structures where the stereochemistry was specified. This set of ligands was prepared with the LigPrep tool implemented in Maestro, using the OPLS2005 force field [60], and all possible ionization states and tautomers at pH 7 ± 1 were generated with Epik [61,62]. Through this procedure, we obtained a total of 146 ligands that were subsequently split into two groups according to the reported MIC values. Specifically, we considered active compounds to be those with an MIC ≤ 1 µg/mL, and inactive compounds to be those with an MIC > 1 µg/mL. Using this threshold, we obtained 83 active molecules and 63 inactive ones.

### 3.4. Docking

All docking calculations were carried out using the Glide software implemented in Maestro [45,46]. The docking protocol consists of the following settings: (a) penalization of non-planar amide conformations; (b) sampling of nitrogen inversions and ring conformations; (c) favoring the π conjugated group’s planarity; (d) adding the calculated Epik state penalties to the final scoring value; (e) considering aromatic hydrogen atoms as H-bond donors, and halogens as H-bond acceptors. All calculations were performed using the standard precision mode. The BEDROC metric [63] was calculated using the enrichment Python script from the Schrödinger suite. In this case, we focused on BEDROC with α = 8.0, i.e., we considered the first 20% of the rank to account for 80% of the score. The ROC curves [64] were obtained with an in-house Python script. 

## 4. Conclusions

In this work, we used classical MD simulations and docking calculations to characterize the key interactions for the binding of novel bacterial topoisomerase inhibitors (NBTIs) to the DNA gyrase enzyme. Together with the crystallographic H-bond (Hb1), which connects Asp83D with the NBTI linker, our MD simulations revealed the possible formation of a second favorable H-bond (Hb2) between Asp83B and the inhibitor (Figure 1). Hb2 is formed via the reorientation of Asp83B, which rotates to point toward the ligand. Inspection of a set of X-ray structures of DNA gyrase and topoisomerase IV revealed a broad distribution of conformations of Asp83B (Table S1). This further supports this residue’s intrinsic flexibility, as demonstrated in our MD simulations, and also explains the conformational switch we observed. Finally, we validated these findings through docking calculations, demonstrating that the formation of Hb2 enhances the enrichment of a dataset of ~150 cognate NBTIs. These results might help in identifying new NBTIs against DNA gyrase. 

## Supplementary Materials

Supplementary materials can be found at http://www.mdpi.com/1422-0067/19/2/453/s1.

## References

[1] Champoux J.J. (2001). DNA topoisomerases: Structure, function, and mechanism. Annu. Rev. Biochem..

[2] Palermo G., Minniti E., Greco M.L., Riccardi L., Simoni E., Convertino M., Marchetti C., Rosini M., Sissi C., Minarini A. (2015). An optimized polyamine moiety boosts the potency of human type II topoisomerase poisons as quantified by comparative analysis centered on the clinical candidate F14512. Chem. Commun..

[3] Chatterji M., Unniraman S., Mahadevan S., Nagaraja V. (2001). Effect of different classes of inhibitors on DNA gyrase from Mycobacterium smegmatis. J. Antimicrob. Chemother..

[4] Rajendram M., Hurley K.A., Foss M.H., Thornton K.M., Moore J.T., Shaw J.T., Weibel D.B. (2014). Gyramides prevent bacterial growth by inhibiting DNA gyrase and altering chromosome topology. ACS Chem. Biol..

[5] Basarab G.S., Hill P.J., Garner C.E., Hull K., Green O., Sherer B.A., Dangel P.B., Manchester J.I., Bist S., Hauck S. (2014). Optimization of pyrrolamide topoisomerase II inhibitors toward identification of an antibacterial clinical candidate (AZD5099). J. Med. Chem..

[6] Minniti E., Byl J.A.W., Riccardi L., Sissi C., Rosini M., de Vivo M., Minarini A., Osheroff N. (2017). Novel xanthone-polyamine conjugates as catalytic inhibitors of human topoisomerase IIα. Bioorg. Med. Chem. Lett..

[7] Ortega J.A., Riccardi L., Minniti E., Borgogno M., Arencibia J.M., Greco M.L., Minarini A., Sissi C., de Vivo M. (2017). Pharmacophore hybridization to discover novel Topoisomerase II poisons with promising antiproliferative activity. J. Med. Chem..

[8] Fisher L.M., Pan X.S. (2008). Methods to assay inhibitors of DNA gyrase and topoisomerase IV activities. Methods Mol. Med..

[9] Nagaraja V., Godbole A.A., Henderson S.R., Maxwell A. (2017). DNA topoisomerase I and DNA gyrase as targets for TB therapy. Drug Discov. Today.

[10] Chan P.F., Srikannathasan V., Huang J., Cui H., Fosberry A.P., Gu M., Hann M.M., Hibbs M., Homes P., Ingraham K. (2015). Structural basis of DNA gyrase inhibition by antibacterial QPT-1, anticancer drug etoposide and moxifloxacin. Nat. Commun..

[11] Heeb S., Fletcher M.P., Chhabra S.R., Diggle S.P., Williams P., Cámara M. (2011). Quinolones: From antibiotics to autoinducers. FEMS Microbiol. Rev..

[12] Aldred K.J., Kerns R.J., Osheroff N. (2014). Mechanism of quinolone action and resistance. Biochemistry.

[13] Aldred K.J., Schwanz H.A., Li G., McPherson S.A., Turnbough C.L., Kerns R.J., Osheroff N. (2013). Overcoming target-mediated quinolone resistance in topoisomerase IV by introducing metal-ion-independent drug-enzyme interactions. ACS Chem. Biol..

[14] Mayer C., Janin Y.L. (2014). Non-quinolone inhibitors of bacterial type IIA topoisomerases: A feat of bioisosterism. Chem. Rev..

[15] Singh S.B. (2014). Confronting the challenges of discovery of novel antibacterial agents. Bioorg. Med. Chem. Lett..

[16] Fournier B., Hooper D.C. (1998). Mutations in topoisomerase IV and DNA gyrase of Staphylococcus aureus: Novel pleiotropic effects on quinolone and coumarin activity. Antimicrob. Agents Chemother..

[17] Basarab G.S., Kern G.H., McNulty J., Mueller J.P., Lawrence K., Vishwanathan K., Alm R.A., Barvian K., Doig P., Galullo V. (2015). Responding to the challenge of untreatable gonorrhea: ETX0914, a first-in-class agent with a distinct mechanism-of-action against bacterial type II topoisomerases. Sci. Rep..

[18] Collin F., Karkare S., Maxwell A. (2011). Exploiting bacterial DNA gyrase as a drug target: Current state and perspectives. Appl. Microbiol. Biotechnol..

[19] Charrier C., Salisbury A.-M., Savage V.J., Duffy T., Moyo E., Chaffer-Malam N., Ooi N., Newman R., Cheung J., Metzger R. (2017). Novel bacterial topoisomerase inhibitors with potent broad-spectrum activity against drug-resistant bacteria. Antimicrob. Agents Chemother..

[20] Kolaric A., Minovski N. (2017). Structure-based design of novel combinatorially generated NBTIs as potential DNA gyrase inhibitors against various Staphylococcus aureus mutant strains. Mol. Biosyst..

[21] Singh S.B., Kaelin D.E., Wu J., Miesel L., Tan C.M., Meinke P.T., Olsen D.B., Lagrutta A., Bradley P., Lu J. (2014). Oxabicyclooctane-linked novel bacterial topoisomerase inhibitors as broad spectrum antibacterial agents. ACS Med. Chem. Lett..

[22] Coates W.J., Gwynn M.N., Hatton I.K., Masters P.J., Pearson N.D., Rahman S.S., Slocombe B., Warrack J.D. (2000). Quinolone Derivatives as Antibacterials.

[23] Bax B.D., Chan P.F., Eggleston D.S., Fosberry A.P., Gentry D.R., Gorrec F., Giordano I., Hann M.M., Hennessy A.J., Hibbs M. (2010). Type IIA topoisomerase inhibition by a new class of antibacterial agents. Nature.

[24] Singh S.B., Kaelin D.E., Wu J., Miesel L., Tan C.M., Meinke P.T., Olsen D.B., Lagrutta A., Wei C., Liao Y. (2015). C1-C2-linker substituted 1,5-naphthyridine analogues of oxabicyclooctane-linked NBTIs as broad-spectrum antibacterial agents (part 7). Med. Chem. Commun..

[25] Tan C.M., Gill C.J., Wu J., Toussaint N., Yin J., Tsuchiya T., Garlisi C.G., Kaelin D.E., Meinke P.T., Miesel L. (2016). In vitro and in vivo characterization of the novel oxabicyclooctane-linked bacterial topoisomerase inhibitor AM-8722, a selective, potent inhibitor of bacterial DNA gyrase. Antimicrob. Agents Chemother..

[26] Hameed S.P., Raichurkar A., Madhavapeddi P., Menasinakai S., Sharma S., Kaur P., Nandishaiah R., Panduga V., Reddy J., Sambandamurthy V.K. (2014). Benzimidazoles: Novel mycobacterial gyrase inhibitors from scaffold morphing. ACS Med. Chem. Lett..

[27] Miles T.J., Hennessy A.J., Bax B.D., Brooks G., Brown B.S., Brown P., Cailleau N., Chen D., Dabbs S., Davies D.T. (2013). Novel hydroxyl tricyclics (e.g., GSK966587) as potent inhibitors of bacterial type IIA topoisomerases. Bioorg. Med. Chem. Lett..

[28] Miles T.J., Hennessy A.J., Bax B.D., Brooks G., Brown B.S., Brown P., Cailleau N., Chen D., Dabbs S., Davies D.T. (2016). Novel tricyclics (e.g., GSK945237) as potent inhibitors of bacterial type IIA topoisomerases. Bioorg. Med. Chem. Lett..

[29] Chan P.F., Germe T., Bax B.D., Huang J., Thalji R.K., Bacqué E., Checchia A., Chen D., Cui H., Ding X. (2017). Thiophene antibacterials that allosterically stabilize DNA-cleavage complexes with DNA gyrase. Proc. Natl. Acad. Sci. USA.

[30] Singh S.B., Kaelin D.E., Wu J., Miesel L., Tan C.M., Black T., Nargund R., Meinke P.T., Olsen D.B., Lagrutta A. (2015). Tricyclic 1,5-naphthyridinone oxabicyclooctane-linked novel bacterial topoisomerase inhibitors as broad-spectrum antibacterial agents-SAR of left-hand-side moiety (Part-2). Bioorg. Med. Chem. Lett..

[31] Singh S.B., Kaelin D.E., Meinke P.T., Wu J., Miesel L., Tan C.M., Olsen D.B., Lagrutta A., Fukuda H., Kishii R. (2015). Structure activity relationship of C-2 ether substituted 1,5-naphthyridine analogs of oxabicyclooctane-linked novel bacterial topoisomerase inhibitors as broad-spectrum antibacterial agents (Part-5). Bioorg. Med. Chem. Lett..

[32] Singh S.B., Kaelin D.E., Wu J., Miesel L., Tan C.M., Meinke P.T., Olsen D.B., Lagrutta A., Wei C., Liao Y. (2015). Structure activity relationship of pyridoxazinone substituted RHS analogs of oxabicyclooctane-linked 1,5-naphthyridinyl novel bacterial topoisomerase inhibitors as broad-spectrum antibacterial agents (Part-6). Bioorg. Med. Chem. Lett..

[33] Singh S.B., Kaelin D.E., Wu J., Miesel L., Tan C.M., Gill C.J., Black T., Nargund R., Meinke P.T., Olsen D.B. (2015). Hydroxy tricyclic 1,5-naphthyridinone oxabicyclooctane-linked novel bacterial topoisomerase inhibitors as broad-spectrum antibacterial agents-SAR of RHS moiety (Part-3). Bioorg. Med. Chem. Lett..

[34] Singh S.B., Kaelin D.E., Wu J., Miesel L., Tan C.M., Meinke P.T., Olsen D.B., Lagrutta A., Wei C., Peng X. (2015). Structure activity relationship of substituted 1,5-naphthyridine analogs of oxabicyclooctane-linked novel bacterial topoisomerase inhibitors as broad-spectrum antibacterial agents (Part-4). Bioorg. Med. Chem. Lett..

[35] La Sala G., Riccardi L., Gaspari R., Cavalli A., Hantschel O., De Vivo M. (2016). HRD motif as the central hub of the signaling network for activation loop autophosphorylation in Abl kinase. J. Chem. Theory Comput..

[36] Palermo G., Bauer I., Campomanes P., Cavalli A., Armirotti A., Girotto S., Rothlisberger U., de Vivo M. (2015). Keys to lipid selection in fatty acid amide hydrolase catalysis: Structural flexibility, gating residues and multiple binding pockets. PLoS Comput. Biol..

[37] Palermo G., Rothlisberger U., Cavalli A., De Vivo M. (2015). Computational insights into function and inhibition of fatty acid amide hydrolase. Eur. J. Med. Chem..

[38] Veselkov D.A., Laponogov I., Pan X.S., Selvarajah J., Skamrova G.B., Branstrom A., Narasimhan J., Prasad J.V.N.V., Fisher L.M., Sanderson M.R. (2016). Structure of a quinolone-stabilized cleavage complex of topoisomerase IV from Klebsiella pneumoniae and comparison with a related Streptococcus pneumoniae complex. Acta Crystallogr. Sect. D Struct. Biol..

[39] Piton J., Petrella S., Delarue M., André-Leroux G., Jarlier V., Aubry A., Mayer C. (2010). Structural insights into the quinolone resistance mechanism of Mycobacterium tuberculosis DNA gyrase. PLoS ONE.

[40] Lahiri S.D., Kutschke A., McCormack K., Alm R.A. (2015). Insights into the mechanism of inhibition of novel bacterial topoisomerase inhibitors from characterization of resistant mutants of Staphylococcus aureus. Antimicrob. Agents Chemother..

[41] De Vivo M., Cavalli A. (2017). Recent advances in dynamic docking for drug discovery. Wiley Interdiscip. Rev. Comput. Mol. Sci..

[42] De Vivo M., Masetti M., Bottegoni G., Cavalli A. (2016). Role of molecular dynamics and related methods in drug discovery. J. Med. Chem..

[43] Jacobson M.P., Friesner R.A., Xiang Z., Honig B. (2002). On the Role of the Crystal Environment in Determining Protein Side-chain Conformations. J. Mol. Biol..

[44] Jacobson M.P., Pincus D.L., Rapp C.S., Day T.J.F., Honig B., Shaw D.E., Friesner R.A. (2004). A hierarchical approach to all-atom protein loop prediction. Proteins Struct. Funct. Bioinform..

[45] Halgren T.A., Murphy R.B., Friesner R.A., Beard H.S., Frye L.L., Pollard W.T., Banks J.L. (2004). Glide: A new approach for rapid, accurate docking and scoring. 2. Enrichment factors in database screening. J. Med. Chem..

[46] Friesner R.A., Banks J.L., Murphy R.B., Halgren T.A., Klicic J.J., Mainz D.T., Repasky M.P., Knoll E.H., Shelley M., Perry J.K. (2004). Glide: A new approach for rapid, accurate docking and scoring. 1. Method and assessment of docking accuracy. J. Med. Chem..

[47] Maier J.A., Martinez C., Kasavajhala K., Wickstrom L., Hauser K.E., Simmerling C. (2015). ff14SB: Improving the accuracy of protein side chain and backbone parameters from ff99SB. J. Chem. Theory Comput..

[48] Cornell W.D., Cieplak P., Bayly C.I., Kollmann P.A. (1993). Application of RESP charges to calculate conformational energies, hydrogen bond energies, and free energies of solvation. J. Am. Chem. Soc..

[49] Wang J., Wolf R.M., Caldwell J.W., Kollman P.A., Case D.A. (2004). Development and testing of a general amber force field. J. Comput. Chem..

[50] Jorgensen W.L., Chandrasekhar J., Madura J.D., Impey R.W., Klein M.L. (1983). Comparison of simple potential functions for simulating liquid water. J. Chem. Phys..

[51] Berendsen H.J.C., Postma J.P.M., van Gunsteren W.F., DiNola A., Haak J.R. (1984). Molecular dynamics with coupling to an external bath. J. Chem. Phys..

[52] Parrinello M., Rahman A. (1981). Polymorphic transitions in single crystals: A new molecular dynamics method. J. Appl. Phys..

[53] Hess B., Bekker H., Berendsen H.J.C., Fraaije J.G.E.M. (1997). LINCS: A linear constraint solver for molecular simulations. J. Comput. Chem..

[54] Darden T., York D., Pedersen L. (1993). Particle mesh Ewald: An N⋅log(N) method for Ewald sums in large systems. J. Chem. Phys..

[55] Hess B., Kutzner C., van der Spoel D., Lindahl E. (2008). GROMACS 4: Algorithms for highly efficient, load-balanced, and scalable molecular simulation. J. Chem. Theory Comput..

[56] Palermo G., Campomanes P., Neri M., Piomelli D., Cavalli A., Rothlisberger U., de Vivo M. (2013). Wagging the tail: Essential role of substrate flexibility in FAAH catalysis. J. Chem. Theory Comput..

[57] Riccardi L., Arencibia J.M., Bono L., Armirotti A., Girotto S., de Vivo M. (2017). Lid domain plasticity and lipid flexibility modulate enzyme specificity in human monoacylglycerol lipase. Biochim. Biophys. Acta Mol. Cell Biol. Lipids.

[58] Sastry G.M., Adzhigirey M., Day T., Annabhimoju R., Sherman W. (2013). Protein and ligand preparation: Parameters, protocols, and influence on virtual screening enrichments. J. Comput. Aided Mol. Des..

[59] Olsson M.H.M., Søndergaard C.R., Rostkowski M., Jensen J.H. (2011). PROPKA3: Consistent treatment of internal and surface residues in empirical pKa predictions. J. Chem. Theory Comput..

[60] Banks J.L., Beard H.S., Cao Y., Cho A.E., Damm W., Farid R., Felts A.K., Halgren T.A., Mainz D.T., Maple J.R. (2005). Integrated modeling program, applied chemical theory (IMPACT). J. Comput. Chem..

[61] Shelley J.C., Cholleti A., Frye L.L., Greenwood J.R., Timlin M.R., Uchimaya M. (2007). Epik: A software program for pKa prediction and protonation state generation for drug-like molecules. J. Comput. Aided Mol. Des..

[62] Greenwood J.R., Calkins D., Sullivan A.P., Shelley J.C. (2010). Towards the comprehensive, rapid, and accurate prediction of the favorable tautomeric states of drug-like molecules in aqueous solution. J. Comput. Aided Mol. Des..

[63] Truchon J.F., Bayly C.I. (2007). Evaluating virtual screening methods: Good and bad metrics for the “early recognition” problem. J. Chem. Inf. Model..

[64] Zhao W., Hevener K.E., White S.W., Lee R.E., Boyett J.M. (2009). A statistical framework to evaluate virtual screening. BMC Bioinform..

