# Supplementary material for: Novel Bacterial Topoisomerase Inhibitors Exploit Asp83 and the Intrinsic Flexibility of the DNA Gyrase Binding Site
Source: Int J Mol Sci. 2018 Feb 3;19(2):453. doi: 10.3390/ijms19020453 (PMC5855675; doi:10.3390/ijms19020453)
Supplement: Supplementary file 1 [file ijms-19-00453-s001.pdf]

Novel Bacterial Topoisomerase Inhibitors Exploit Asp83 and the Intrinsic Flexibility of the DNA Gyrase Binding Site

the DNA Gyrase Binding Site Sebastian Franco-Ulloa,[1,2,§] Giuseppina La Sala,[2,§]
Gian Pietro Miscione,[*,1] and Marco De Vivo[*,2,3]

1. COBO Computational Bio-Organic Chemistry Bogotá, Chemistry Department, Universidad de los Andes, Cra 1 No 18A-12, 111711 Bogotá, Colombia

2. Molecular Modeling and Drug Discovery Lab, Istituto Italiano di Tecnologia, via Morego 30, 16163 Genova, Italy

3. IAS-S/INM-9 Computational Biomedicine Forschungszentrum Jülich Wilhelm-Johnen-Straße 52428 Jülich, Germany

Corresponding authors:

Dr. Gian Pietro Miscione – Email: gp.miscione57@uniandes.edu.co
Dr. Marco De Vivo – Email: marco.devivo@iit.it

[§]Equally contributed

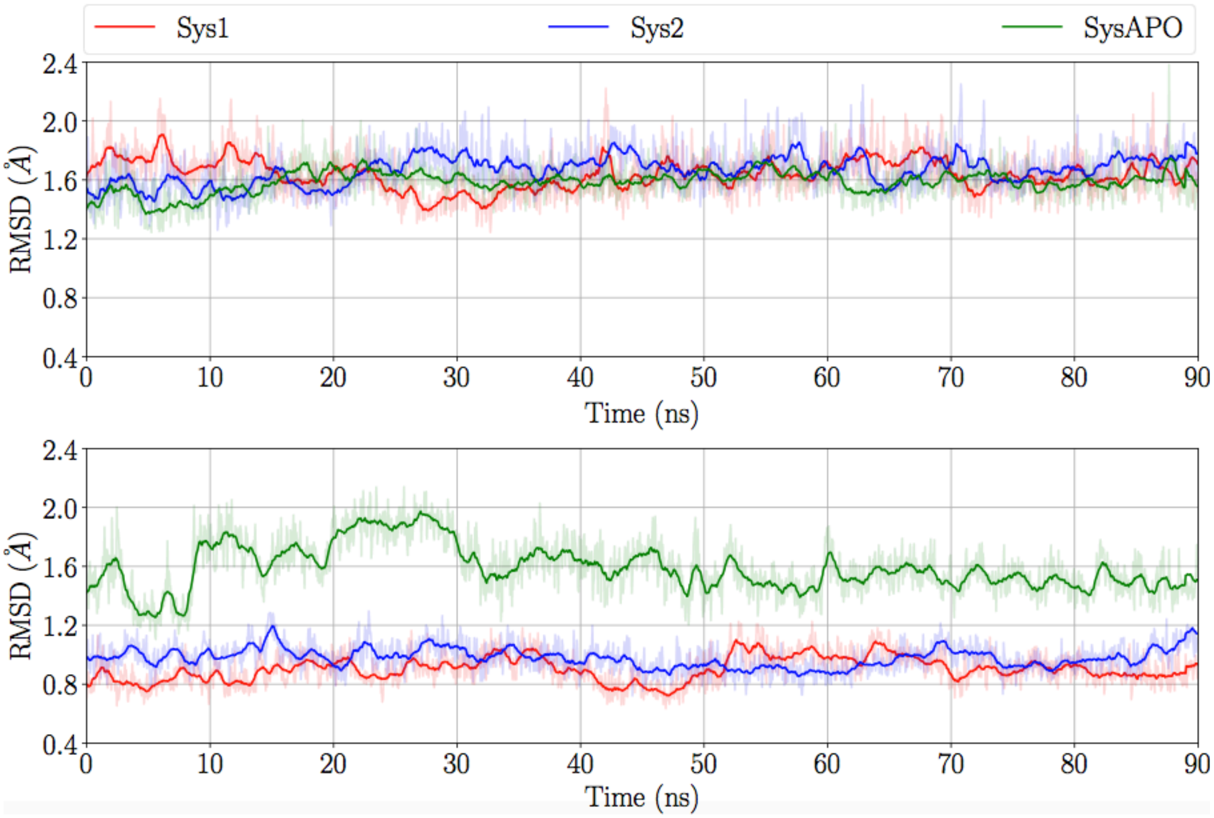

Figure S1. RMSD values of Sys1 (red line), Sys2 (blue line), and SysAPO (green line) of the Cα of the protein (top panel) and of the backbone of the DNA (bottom panel). The RMSDs have been computed using the X-ray structure 4PLB as reference.

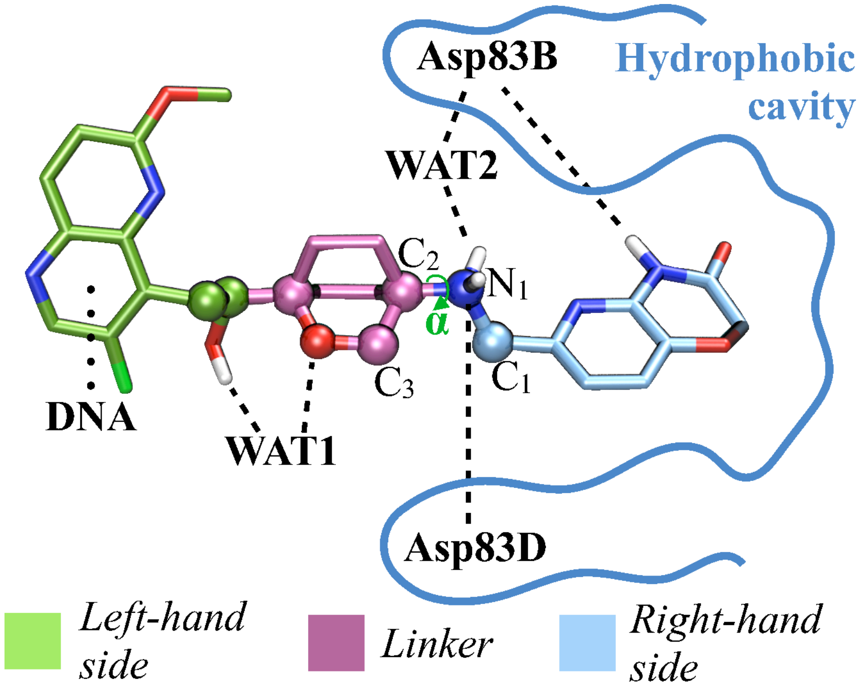

Figure S2. Binding mode of Cpd2. The ligand is divided in three parts: i) the left-hand side (LHS), which interacts with the DNA, ii) the linker, and iii) the right-hand side (RHS) that binds the NBTIs to the protein's pocket through hydrophobic contacts. WAT1 interacts with both the hydroxyl group of the LHS and with the oxabicyclooctane linker. Conversely, WAT2 mediates the H-bond between the Asp83B and the basic nitrogen of the linker.

Table S1. The table lists 43 PDB X-ray structures of both DNA gyrase and Topoisomerase IV of different bacterial strains. Only the structures with resolved Asp83 have been selected. The structures have been divided into three groups depending on the orientation of the Asp83B: i) *4PLB-like*, which includes all the structures having the Asp83B in a conformation similar to the simulated crystal structure 4PLB. In this conformation, the novel H-bond Hb2 is prevented. ii) *MD-like*, which includes all the structures having the Asp83B in a conformation similar to that observed in our MD simulations. Here the aspartate can form the novel H-bond Hb2 with the ligand. iii) *Other*, which includes the Asp83B conformations that do not belong neither to i) nor to ii).

PDB code	Organism	Protein	Asp conformation	Asp Number
2XCS	*S. aureus*	Gyrase	4PLB-like	83
2XCR	*S. aureus*	Gyrase	4PLB-like	83
4BUL	*S. aureus*	Gyrase	4PLB-like	83
5BS3	*S. aureus*	Gyrase	4PLB-like	83
5NPP	*S. aureus*	Gyrase	4PLB-like	83
5IWI	*S. aureus*	Gyrase	4PLB-like	83
5IWM	*S. aureus*	Gyrase	4PLB-like	83
5CDN	*S. aureus*	Gyrase	4PLB-like	83
5CDP	*S. aureus*	Gyrase	MD-like	83
5CDQ	*S. aureus*	Gyrase	4PLB-like	83
5NPK	*S. aureus*	Gyrase	4PLB-like	83
5CDM	*S. aureus*	Gyrase	4PLB-like	83
2XCT	*S. aureus*	Gyrase	MD-like	83
5CDO	*S. aureus*	Gyrase	4PLB-like	83
5CDR	*S. aureus*	Gyrase	4PLB-like	83
4Z2C	*S. pneumoniae*	Gyrase	Other	80
4Z2D	*S. pneumoniae*	Gyrase	Other	80
4Z2E	*S. pneumoniae*	Gyrase	Other	80
5BS8	*M. tuberculosis*	Gyrase	4PLB-like	89
5BTA	*M. tuberculosis*	Gyrase	4PLB-like	89
5BTC	*M. tuberculosis*	Gyrase	4PLB-like	89
5BTD	*M. tuberculosis*	Gyrase	4PLB-like	89
5BTF	*M. tuberculosis*	Gyrase	4PLB-like	89
5BTG	*M. tuberculosis*	Gyrase	4PLB-like	89
5BTI	*M. tuberculosis*	Gyrase	4PLB-like	89

5BTL	*M. tuberculosis*	Gyrase	4PLB-like	89
5BTN	*M. tuberculosis*	Gyrase	4PLB-like	89
3IFZ	*M. tuberculosis*	Gyrase	Other	89
3LTN	*S. pneumoniae*	Topo IV	4PLB-like	78
3RAF	*S. pneumoniae*	Topo IV	4PLB-like	78
4Z3O	*S. pneumoniae*	Topo IV	4PLB-like	78
4Z53	*S. pneumoniae*	Topo IV	4PLB-like	78
4Z4Q	*S. pneumoniae*	Topo IV	4PLB-like	78
5EIX	*K. pneumoniae*	Topo IV	MD-like	79
3RAD	*S. pneumoniae*	Topo IV	4PLB-like	78
3RAE	*S. pneumoniae*	Topo IV	4PLB-like	78
3KPF	*S. pneumoniae*	Topo IV	4PLB-like	78
4KOE	*S. pneumoniae*	Topo IV	4PLB-like	78
4KPE	*S. pneumoniae*	Topo IV	4PLB-like	78
4KPF	*S. pneumoniae*	Topo IV	4PLB-like	78
2XKK	*A. baumannii*	Topo IV	4PLB-like	83
3KSA	*S. pneumoniae*	Topo IV	MD-like	78
3KSB	*S. pneumoniae*	Topo IV	4PLB-like	78
